# From genes to clinical management: A comprehensive review of long QT syndrome pathogenesis and treatment

**DOI:** 10.1016/j.hroo.2024.07.006

**Published:** 2024-07-15

**Authors:** Wenjing Zhu, Xueyan Bian, Jianli Lv

**Affiliations:** ∗ Department of Pulmonary and Critical Care Medicine, Shandong Provincial Hospital Affiliated to Shandong First Medical University, Jinan, Shandong, China; † Department of Pediatrics, Lixia District People’s Hospital, Jinan, Shandong, China; ‡ Department of Pediatric Cardiology, Shandong Provincial Hospital Affiliated to Shandong First Medical University, Jinan, Shandong, China

**Keywords:** Long QT syndrome, Ventricular arrhythmias, Ion channelopathies, Diagnosis, Treatment strategies

## Abstract

**Background:**

Long QT syndrome (LQTS) is a rare cardiac disorder characterized by prolonged ventricular repolarization and increased risk of ventricular arrhythmias. This review summarizes current knowledge of LQTS pathogenesis and treatment strategies.

**Objectives:**

The purpose of this study was to provide an in-depth understanding of LQTS genetic and molecular mechanisms, discuss clinical presentation and diagnosis, evaluate treatment options, and highlight future research directions.

**Methods:**

A systematic search of PubMed, Embase, and Cochrane Library databases was conducted to identify relevant studies published up to April 2024.

**Results:**

LQTS involves mutations in ion channel–related genes encoding cardiac ion channels, regulatory proteins, and other associated factors, leading to altered cellular electrophysiology. Acquired causes can also contribute. Diagnosis relies on clinical history, electrocardiographic findings, and genetic testing. Treatment strategies include lifestyle modifications, β-blockers, potassium channel openers, device therapy, and surgical interventions.

**Conclusion:**

Advances in understanding LQTS have improved diagnosis and personalized treatment approaches. Challenges remain in risk stratification and management of certain patient subgroups. Future research should focus on developing novel pharmacological agents, refining device technologies, and conducting large-scale clinical trials. Increased awareness and education are crucial for early detection and appropriate management of LQTS.

**Key Findings**

1. Pathogenesis
  ▪ Long QT syndrome (LQTS) involves mutations in ion channel–related genes, leading to altered cellular electrophysiology and prolonged ventricular repolarization.
  ▪ Both congenital and acquired forms of LQTS exist, with the former being genetically heterogeneous and the latter often drug induced or due to electrolyte imbalances.
2. Diagnosis
  ▪ Diagnosis of LQTS relies on a combination of clinical history, electrocardiographic findings, and genetic testing.
  ▪ The Schwartz score and genetic testing are critical tools for assessing the probability of LQTS and identifying specific subtypes.
3. Epidemiology and clinical importance
  ▪ The prevalence of congenital LQTS is estimated to be 1:2000 to 1:2500, but the actual prevalence may be higher because of silent mutation carriers.
  ▪ LQTS is a significant cause of sudden cardiac death in young, apparently healthy individuals.
4. Historical perspective
  ▪ LQTS has been recognized since the 1950s, with significant advancements in understanding its genetic basis and clinical management over the decades.
  ▪ The identification of key genes (*KCNQ1*, *KCNH2*, and *SCN5A*) in the 1990s was a major breakthrough in understanding the disorder.
5. Treatment strategies
  ▪ Treatment includes lifestyle modifications, β-blockers, potassium channel openers, device therapy, and surgical interventions.
  ▪ Advances in understanding LQTS have led to personalized treatment approaches, improving patient care and outcomes.
6. Future directions
  ▪ Future research should focus on developing novel pharmacological agents, refining device technologies, and conducting large-scale clinical trials.
  ▪ Increased awareness and education are crucial for early detection and appropriate management of LQTS.

## Introduction

### Definition of long QT syndrome

Long QT syndrome (LQTS) is a genetically heterogeneous cardiac channelopathy characterized by prolonged ventricular repolarization, which manifests as a prolonged QT interval on the surface electrocardiogram (ECG).^1^ The prolonged QT interval results from delayed inactivation of inward sodium (I_Na_) or calcium (I_Ca_) currents or a loss of function in outward potassium (slow delayed rectifier potassium channel [I_Ks_], rapid delayed rectifier potassium channel [I_Kr_], or inward rectifier potassium current [I_K1_]) currents, leading to an extended action potential duration (APD) in cardiac myocytes.^2^^,^^3^ This prolonged repolarization predisposes individuals to ventricular arrhythmias, particularly torsades de pointes (TdP), a polymorphic ventricular tachycardia that can degenerate into ventricular fibrillation and cause sudden cardiac death (SCD).^4^

LQTS can be classified into 2 main categories: congenital and acquired. Congenital LQTS is caused by genetic mutations in genes encoding cardiac ion channels or their regulatory proteins, resulting in altered channel function and prolonged repolarization.^5^ Mutations in at least 17 genes have been associated with LQTS, with LQTS type 1 (LQT1) (*KCNQ1* gene), LQTS type 2 (LQT2) (*KCNH2* gene), and LQTS type 3 (LQT3) (*SCN5A* gene) accounting for ∼80%–90% of genotype-positive cases.^6^^,^^7^ Conversely, acquired LQTS (aLQTS) results from various environmental factors, including certain medications (eg, antiarrhythmic drugs, antibiotics, and antipsychotics), electrolyte imbalances (eg, hypokalemia and hypomagnesemia), and medical conditions (eg, bradycardia and hypothyroidism).^8^^,^^9^

Diagnosis of LQTS relies on a comprehensive evaluation of clinical history, family history, ECG findings, and genetic testing. The Schwartz score, which incorporates these factors, is widely used to assess the probability of LQTS, with a score of ≥3.5 indicating a high likelihood of the disorder.^10^ Genetic testing has emerged as a crucial tool for confirming the diagnosis and identifying specific LQTS subtypes, which informs risk stratification and management strategies.^11^

### Epidemiology and clinical importance

The prevalence of congenital LQTS is estimated to be ∼1:2000 to 1:2500 in the general population.^3^^,^^12^^,^^13^ However, the actual prevalence may be higher because of variable penetrance and expressivity as well as the presence of silent mutation carriers.^14^ The prevalence of aLQTS is more challenging to determine, as it depends on exposure to various environmental triggers.^15^

LQTS is clinically significant because of its potential to cause life-threatening ventricular arrhythmias, particularly TdP, which can lead to syncope, seizures, and SCD.^4^ It is a leading cause of SCD in young, apparently healthy individuals, accounting for ∼54% of sudden unexplained deaths in those younger than 35 years.^16^ Clinical presentation varies widely, from asymptomatic individuals to those experiencing recurrent syncope or cardiac arrest.^17^ The risk of life-threatening arrhythmias is influenced by factors such as age, sex, genotype, QT interval, and exposure to triggers.^18^

The importance of LQTS extends to family members. Given the autosomal dominant inheritance pattern of the most common subtypes (LQT1, LQT2, and LQT3), first-degree relatives of an affected individual have a 50% chance of carrying the pathogenic variant.^19^ Cascade genetic screening is crucial for identifying asymptomatic carriers who may benefit from preventive measures and close monitoring.^20^

Furthermore, LQTS serves as a paradigm for understanding the complex interplay between genetic predisposition and environmental factors in cardiac arrhythmias.^21^ Insights gained from studying LQTS have improved our understanding of the disorder and contributed to the development of targeted therapies and risk stratification strategies for other cardiac channelopathies and arrhythmogenic disorders.^22^

### Historical perspective

The first description of LQTS dates back to 1957 when Jervell and Lange-Nielsen reported a family with congenital deafness, prolonged QT interval, and sudden death.^23^ This autosomal recessive form, now known as Jervell and Lange-Nielsen syndrome (JLNS), was later found to be caused by homozygous or compound heterozygous mutations in the *KCNQ1* or *KCNE1* genes, encoding subunits of the I_Ks_.^24^

In 1963 and 1964, Romano^25^ and Ward^26^ independently described families with a prolonged QT interval and syncope without deafness, which became known as Romano-Ward syndrome. Romano-Ward syndrome, an autosomal dominant form of LQTS, is more common than JLNS. Studies in the 1970s and 1980s further characterized the clinical features and familial nature of LQTS, leading to the recognition of its genetic heterogeneity.^27^

A major breakthrough occurred in the 1990s with the identification of the first 3 LQTS-associated genes: *KCNQ1* (LQT1), *KCNH2* (LQT2), and *SCN5A* (LQT3).^28^ These discoveries improved understanding of LQTS pathophysiology and paved the way for genotype-specific management strategies.^29^

Since then, numerous other genes have been implicated in LQTS, with at least 17 genes currently associated with the disorder.^30^ These genes encode various ion channels, channel subunits, and regulatory proteins involved in cardiac repolarization. The expanding genetic knowledge has led to more comprehensive testing panels and facilitated genotype-phenotype correlations.^31^

Advancements in clinical management have paralleled genetic discoveries. β-Blockers, established as the mainstay of LQTS therapy in the 1970s, remain a cornerstone of treatment.^32^ Implantable cardioverter-defibrillators (ICDs) have been increasingly used since the 1990s for high-risk patients or those with recurrent events despite medical therapy.^33^ More recently, left cardiac sympathetic denervation (LCSD) has emerged as an effective adjunctive therapy for patients with refractory symptoms or β-blocker intolerance.^34^

The historical journey of LQTS, from its initial description to the current understanding of its genetic basis and clinical management, has been marked by significant advancements in molecular genetics, electrophysiology, and clinical cardiology. These advancements have improved patient care and provided valuable insights into the complex mechanisms underlying cardiac arrhythmogenesis.

## Methods

### Literature search and study selection

This review was conducted in accordance with the Preferred Reporting Items for Systematic Reviews and Meta-Analyses guidelines. A comprehensive literature search was performed using databases such as PubMed, EMBASE, and Cochrane Library to identify relevant studies on LQTS. Keywords and MeSH terms related to LQTS were used to ensure an extensive search. Studies discussing the pathogenesis, diagnosis, treatment, or epidemiology of LQTS were included.

### Pathogenesis of LQTS

#### Genetic basis and molecular mechanisms of LQTS

LQTS is a genetically heterogeneous disorder, with at least 17 genes implicated in its pathogenesis.^35^ These genes primarily encode cardiac ion channels or their regulatory proteins, leading to altered ion channel function, prolonged ventricular repolarization, and increased arrhythmia risk.^36^

The 3 most common LQTS subtypes are LQT1, LQT2, and LQT3, accounting for ∼80%–90% of genotype-positive cases.^35^ LQT1, the most prevalent subtype, is caused by loss-of-function mutations in the *KCNQ1* gene, encoding the α subunit of the I_Ks_.^37^ LQT2 results from loss-of-function mutations in the *KCNH2* gene (also known as human Ether-à-go-go-related gene), encoding the α subunit of the I_Kr_.^38^ LQT3 is caused by gain-of-function mutations in the *SCN5A* gene, encoding the α subunit of the cardiac sodium channel (Nav1.5).^39^

Other less common LQTS subtypes include mutations in genes such as *ANKB*, *KCNE1*, *KCNE2*, *KCNJ2*, and *CACNA1C*,^40^, ^41^, ^42^, ^43^, ^44^, ^45^ which encode various ion channels and their regulatory proteins. Mutations in these genes lead to similar mechanisms of prolonged repolarization and increased arrhythmia risk.

Genetic mutations associated with LQTS lead to ion channelopathies, disorders characterized by abnormal ion channel function at the molecular level. These channelopathies disrupt the delicate balance of ionic currents responsible for proper cardiac myocyte repolarization, resulting in prolonged APD and increased arrhythmia risk.^46^

*LQT1:* Loss-of-function mutations in *KCNQ1* reduce I_Ks_, a key repolarizing current that activates slowly and contributes to action potential termination. Reduced I_Ks_ leads to prolonged APD and QT interval.^47^
*LQT2:* Loss-of-function mutations in *KCNH2* reduce I_Kr_, another critical repolarizing current that activates rapidly and helps terminate the action potential.^38^^,^^48^
*LQT3:* Gain-of-function mutations in *SCN5A* increase the late sodium current (I_Na__L_), leading to a persistent I_Na_ during the plateau phase, prolonging APD and QT interval.^49^

Understanding these mechanisms provides insight into the pathophysiology of LQTS and guides the development of targeted therapies and risk stratification strategies.

#### Acquired causes of LQTS

##### Drugs and substances that can prolong the QT interval

aLQTS occurs when the QT interval is prolonged because of external factors such as medications, illicit substances, or toxins without a genetic predisposition. The most common cause of aLQTS is drug-induced QT prolongation, which is a significant reason for drug withdrawal or relabeling.^50^

Various medications across multiple therapeutic classes have been linked to QT prolongation and an increased risk of TdP. Notable examples include the following:

*Antiarrhythmic drugs:* class IA (eg, quinidine, procainamide, and disopyramide) and class III (eg, sotalol, dofetilide, and ibutilide)^51^
*Antibiotics:* Macrolides (eg, erythromycin, clarithromycin, and azithromycin) and fluoroquinolones (eg, levofloxacin and moxifloxacin)^15^
*Antipsychotics:* Typical (eg, haloperidol and thioridazine) and atypical (eg, risperidone, quetiapine, and ziprasidone)^52^^,^^53^
*Antidepressants:* Tricyclic antidepressants (eg, amitriptyline and imipramine) and selective serotonin reuptake inhibitors (eg, citalopram and fluoxetine)^54^
*Antihistamines:* Nonsedating antihistamines, such as terfenadine and astemizole (both withdrawn)^55^
*Antimalarials:* Chloroquine and hydroxychloroquine, particularly with azithromycin^56^
*Antifungals:* Azole antifungals (eg, ketoconazole and fluconazole)^57^
*Antiemetics:* 5-hydroxytryptamine type 3 receptor antagonists (eg, ondansetron and granisetron)^58^
*Opioids:* Methadone, used for opioid addiction treatment and chronic pain management^59^

In addition to medications, *illicit substances* such as cocaine and methamphetamine have been associated with QT prolongation and increased risk of SCD.^60^^,^^61^

The risk of drug-induced LQTS depends on several factors, including the drug’s inherent proarrhythmic potential, dosage, route of administration, pharmacokinetic properties, and individual patient characteristics (eg, age, sex, comorbidities, electrolyte abnormalities, and genetic predisposition).^62^^,^^63^ Careful evaluation of the risk-benefit ratio, consideration of alternative therapies, and close monitoring are essential when prescribing QT-prolonging medications.

##### Electrolyte imbalances

Electrolyte imbalances, particularly hypokalemia and hypomagnesemia, are well-recognized risk factors for aLQTS and can exacerbate the effects of QT-prolonging drugs.^64^

*Hypokalemia*, defined as serum potassium below 3.5 mEq/L, can lead to prolonged repolarization and increased risk of TdP.^65^ Low extracellular potassium reduces the conductance of the I_K1_, essential for maintaining resting membrane potential and terminal repolarization of cardiac myocytes. It also enhances Na^+^/Ca^2+^ exchanger activity, increasing intracellular calcium levels and prolonging APD.^66^

*Hypomagnesemia*, defined as serum magnesium below 1.7 mg/dL, is another important risk factor for aLQTS.^67^ Magnesium is a critical cofactor for various ion channels and transporters involved in cardiac repolarization. Low magnesium levels can impair the function of I_Kr_, I_Ks_, and Na^+^/K^+^-ATPase, leading to prolonged APD and increased arrhythmia risk.^68^

The combination of hypokalemia and hypomagnesemia can have a synergistic effect on aLQTS and TdP risk.^69^ This is particularly relevant in patients treated with diuretics, which can cause both potassium and magnesium depletion. Other conditions leading to electrolyte imbalances include gastrointestinal disorders, renal disorders, and endocrine disorders.^70^

Hypocalcemia has also been associated with QT prolongation, although the mechanisms are less well understood.^71^ Calcium plays a crucial role in cardiac electrophysiology, and low extracellular calcium levels can alter the function of various ion channels and transporters involved in repolarization.

Identifying and correcting electrolyte imbalances is essential in managing patients with aLQTS or those at risk. Monitoring serum electrolyte levels, particularly in patients treated with QT-prolonging medications or those with predisposing conditions, can help prevent life-threatening arrhythmias.^72^

##### Other medical conditions contributing to aLQTS

Several medical conditions can contribute to the development of aLQTS by directly affecting cardiac repolarization or indirectly influencing the risk of QT prolongation through altered pharmacokinetics or pharmacodynamics of QT-prolonging medications.

*Cardiac conditions*
*Bradycardia:* Slow heart rates can prolong the QT interval and increase TdP risk.^73^^,^^74^
*Heart failure:* Associated with an increased risk of QT prolongation and TdP because of altered ion channel expression, neurohumoral activation, and electrolyte disturbances.^75^
*Myocardial ischemia and infarction:* Can lead to QT prolongation by altering ion channel function and increasing repolarization dispersion.^76^
*Endocrine disorders*
*Hypothyroidism:* Can prolong the QT interval by altering cardiac ion channel expression and function.^77^
*Pheochromocytoma:* Catecholamine excess can lead to QT prolongation and increased ventricular arrhythmia risk.^78^
*Neurological conditions*
*Subarachnoid hemorrhage:* Increased risk of QT prolongation and TdP because of autonomic dysfunction, electrolyte disturbances, and QT-prolonging medications.^79^
*Stroke:* Acute stroke, particularly involving the insular cortex, can lead to QT prolongation and increased ventricular arrhythmia risk.^80^
*Liver disease:* Hepatic dysfunction can alter QT-prolonging drug metabolism, leading to increased plasma levels and higher aLQTS risk.^81^
*Renal disease:* Chronic kidney disease and end-stage renal disease are associated with increased QT prolongation and TdP risk.^82^
*Eating disorders:* Anorexia nervosa and bulimia nervosa can lead to QT prolongation and increased SCD risk.^83^
*Autoimmune disorders:* Systemic lupus erythematosus and other autoimmune disorders have been associated with QT prolongation.^84^, ^85^, ^86^
Recognizing and managing these underlying medical conditions is crucial for minimizing aLQTS risk. Patients with these conditions should be closely monitored for QT prolongation, especially when treated with QT-prolonging medications, and appropriate interventions should be implemented to correct modifiable risk factors.

#### Pathophysiological mechanisms

##### Cellular and cardiac electrophysiology

Cellular and cardiac electrophysiology are crucial for understanding LQTS pathophysiology. The prolonged QT interval results from alterations in the delicate balance of ionic currents governing cardiac APD.^63^ Action potentials are generated by coordinated opening and closing of voltage-gated sodium, calcium, and potassium channels.^87^

The cardiac action potential consists of the following:

1. *Phase 0 (Initial depolarization):* Rapid sodium influx (I_Na_)
2. *Phase 1 (Early repolarization):* Transient outward potassium current
3. *Phase 2 (Plateau):* Balance between inward L-type calcium current (I_CaL_) and outward delayed rectifier potassium currents (I_Kr_ and I_Ks_)
4. *Phase 3 (Final repolarization):* I_K1_

In LQTS, mutations lead to reduced outward potassium currents or increased I_Na_/I_Ca_, prolonging APD and QT interval.^88^ This delayed repolarization increases the risk of early afterdepolarizations (EADs), which can trigger ventricular arrhythmias, particularly TdP.^63^^,^^89^^,^^90^

EAD formation mechanisms involve L-type calcium channel reactivation during prolonged APD^91^ and intracellular calcium overload activating the sodium-calcium exchanger.^92^

Arrhythmogenic substrate in LQTS is modulated by the following:

1. *Adrenergic stimulation:* Enhances I_CaL_ and promotes calcium overload^93^
2. *Bradycardia:* Exacerbates QT prolongation and increases TdP risk^94^
3. *Electrolyte imbalances:* Alter potassium channel and ion transporter function^95^

Understanding LQTS cellular and cardiac electrophysiology has important clinical implications for risk stratification and management. Genotype-specific triggers and QT interval dynamics assessment guide preventive strategies and optimize treatment.^96^ Insights into molecular mechanisms have led to targeted therapies, such as I_Na__L_ inhibitors and potassium channel openers, aiming to correct electrophysiological abnormalities and reduce life-threatening arrhythmia risk.^97^

##### Clinical implications of altered repolarization

Altered repolarization in LQTS has profound clinical implications, creating a substrate highly susceptible to life-threatening ventricular arrhythmias.^98^ The delayed repolarization in LQTS, resulting from the altered function of cardiac ion channels, leads to a prolonged APD and an increased dispersion of repolarization across the ventricular myocardium.^99^ This spatial heterogeneity of repolarization creates a favorable milieu for the generation and propagation of EADs and triggered activity.^100^ EADs, secondary depolarizations during the plateau or repolarization phases, are a hallmark of LQTS and the primary triggering mechanism for TdP.^38^

The risk of EADs and TdP is modulated by genetic, environmental, and physiological factors.^88^ Genotype-specific differences contribute to variable clinical expressivity and arrhythmogenic risk. For example, patients with LQT1, are particularly susceptible to arrhythmias during exercise or emotional stress, as the impaired I_Ks_ function limits the ability to shorten the QT interval during adrenergic activation.^101^

Additional clinical implications include T-wave alternans (TWA) and impaired QT interval rate adaptation. TWA is a marker of electrical instability and predictor of arrhythmic risk.^102^^,^^103^ The impaired QT adaptation, quantified by the QT/RR slope, has been associated with an increased risk of cardiac events.^18^^,^^104^

### Clinical presentation and diagnosis

#### Clinical manifestations of LQTS

The clinical manifestations of LQTS are diverse and can vary significantly among affected individuals, even those with the same genetic subtype.^105^ The hallmark feature is QT interval prolongation on the ECG, reflecting delayed ventricular repolarization.^106^ However, clinical presentation extends beyond ECG findings and includes symptoms related to increased ventricular arrhythmia risk.^107^

Common clinical manifestations are as follows:

*Syncope:* Occurs in ∼60% of untreated patients.^108^ Triggers vary by genetic subtype:
1. *LQT1 (KCNQ1 mutations):* Exercise or swimming, particularly in younger patients^109^
2. *LQT2 (KCNH2 mutations):* Rest or sleep, often in response to auditory triggers^110^
3. *LQT3 (SCN5A mutations):* Sleep or rest, more pronounced at slower heart rates.^110^
*Aborted cardiac arrest or SCD:* SCD can be the presenting symptom in up to 10% of untreated patients.^111^ Risk factors include the following^111^, ^112^, ^113^, ^114^:
1. History of syncope
2. Corrected QT (QTc) interval > 500 ms
3. Family history of SCD
4. History of aborted cardiac arrest
5. Syncope during the first year of life
*Seizures or seizure-like activity:* LQTS can be misdiagnosed as epilepsy.^115^, ^116^, ^117^ Distinction is crucial because of different treatment strategies and prognoses.^118^^,^^119^
*Extracardiac manifestations:* JLNS presenting as congenital bilateral sensorineural deafness in addition to prolonged QT interval.^120^^,^^121^
*Age-specific presentations:* Functional 2:1 atrioventricular block (pseudo-atrioventricular block) due to extreme QT prolongation in neonates and infants.^122^^,^^123^

#### Risk factors for arrhythmia in patients with LQTS

The risk of arrhythmic events in patients with LQTS is influenced by a complex interplay of genetic, clinical, and environmental factors.^124^ Major risk factors include the following:

*Genetic subtype:*
1. LQT2 and LQT3: Higher risk of syncope and SCD compared to LQT1^125^
2. LQT1: High risk during exercise, especially swimming^126^
3. LQT2: Prone to events during rest or sleep^127^
4. LQT3: Higher risk during sleep or at rest.^110^
*QTc interval:* QTc interval > 500 ms is associated with a significantly increased risk of SCD, particularly in patients with LQT1 and LQT2.^105^ In addition, the presence of QTc interval > 500 ms in the first year of life is a major risk factor for SCD in infants with LQTS.^114^
*Sex:* In childhood, males have a higher risk of SCD than do females, particularly in LQT1.^128^ However, after puberty, the risk of SCD is higher in females, especially in LQT2.^129^, ^130^, ^131^
*History of syncope:* A history of syncope is a major risk factor for SCD in patients with LQTS, particularly if the syncope is recurrent or occurs in the absence of a trigger.^132^ In patients with a history of syncope, the risk of SCD is highest in the first year after the syncopal event and decreases thereafter.^132^ The presence of syncope in the first year of life is also a strong predictor of SCD in infants with LQTS.^133^
*Family history of SCD:* Significant risk factor, especially in young relatives (<40 years) or multiple family members.^134^
*Genotype-specific triggers:* The risk of arrhythmic events in patients with LQTS is influenced by genotype-specific triggers, which can precipitate syncope or SCD in susceptible individuals.^135^
*Electrolyte imbalances:* Electrolyte imbalances, particularly hypokalemia and hypomagnesemia, can prolong the QT interval and increase the risk of arrhythmic events.^136^
*Medications:* QT-prolonging drugs increase the arrhythmic risk.^69^^,^^137^
*Comorbidities:* Hypothyroidism, eating disorders, diabetes, obesity, and obstructive sleep apnea.^83^^,^^138^
*Genotype-phenotype modifiers:* In addition to the primary genetic defect, other genetic factors (such as single nucleotide polymorphisms or modifier genes) can influence the clinical manifestations and arrhythmic risk in patients with LQTS. For example, the presence of certain polymorphisms in the *NOS1AP* gene (which encodes a nitric oxide synthase adaptor protein) has been associated with an increased risk of SCD in patients with LQTS, particularly those with LQT1.^139^^,^^140^

#### Diagnostic criteria and tools

##### ECG findings

The hallmark ECG feature of LQTS is a prolonged QT interval, reflecting delayed ventricular repolarization.^141^ QTc interval > 460 ms in females and QTc interval > 450 ms in males are considered prolonged.^142^ However, up to 25% of patients with genetically confirmed LQTS may have normal QTc intervals.^143^^,^^144^ Additional ECG findings suggestive of LQTS include TWA, T-wave notching, and bradycardia.^145^

##### Schwartz score

The Schwartz score assigns points to clinical and ECG features to estimate LQTS probability.^10^ Scores range from 0 to 9, with ≥3.5 indicating high LQTS probability. The clinical features included in the Schwartz score are syncope (1–2 points), congenital deafness (0.5 points), and a family history of LQTS or unexplained sudden death (0.5–1 points). The ECG features included are QTc interval ≥ 480 ms (3 points), 460–479 ms (2 points), or 450–459 ms (1 point); TWA (1 point); notched T waves in 3 leads (1 point); and bradycardia (0.5 points).

##### Genetic testing

Genetic testing is essential for confirming LQTS diagnosis and identifying specific subtypes.^146^ Commercial panels screen for mutations in up to 17 LQTS-associated genes, with a 50%–80% diagnostic yield.^147^^,^^148^ *KCNQ1* (LQT1), *KCNH2* (LQT2), and *SCN5A* (LQT3) account for 75% of genetically confirmed cases.^149^ Genetic testing aids in identifying asymptomatic family members at risk and guiding genotype-specific management.^150^

##### Provocative testing

Provocative tests can unmask LQTS in patients with borderline QTc intervals or normal ECGs^151^:

1. *Exercise stress testing:* Useful for diagnosing LQT1^152^^,^^153^
2. *Epinephrine infusion:* Helps distinguish between LQT1 and LQT2^154^
3. *Mental stress testing:* Potential method for unmasking concealed LQTS^155^

##### Holter monitoring and event recorders

Ambulatory ECG monitoring can capture transient QT prolongation or T-wave abnormalities.^156^^,^^157^ Implantable loop recorders may be used for long-term monitoring or risk stratification.^158^

##### Family screening

Family screening is essential because of the autosomal dominant inheritance of most LQTS subtypes.^159^ First-degree relatives have a 50% chance of carrying the same mutation and should undergo clinical evaluation, ECG screening, and genetic testing.^160^ Cascade screening helps identify asymptomatic carriers who may benefit from preventive measures and regular follow-up.^161^

### Treatment options for LQTS

#### Risk stratification and lifestyle modifications

Effective LQTS management relies on accurate risk stratification and appropriate lifestyle modifications to minimize life-threatening arrhythmic events.^124^ Risk stratification integrates clinical, ECG, and genetic factors to identify patients at highest risk of SCD who may benefit from more aggressive interventions.^125^

#### Identification of high-risk patients

Key risk factors for SCD in patients with LQTS include the following:

1. *QTc interval:* QTc interval ≥ 500 ms is a major risk factor, particularly in LQT1 and LQT2.^162^
2. *Genetic subtype:* Patients with LQT2 have a higher SCD risk compared to other subtypes.^163^
3. *Sex and age:* Males have higher risk in childhood and females after puberty.^130^^,^^164^
4. *History of syncope:* Particularly if recurrent or without trigger.^134^
5. *Family history of SCD:* Especially in young first-degree relatives.^113^

##### Counseling on lifestyle changes and avoidance of triggers

Lifestyle modifications are crucial in LQTS management and should be tailored to the patient’s specific genetic subtype and risk profile.^165^ Key recommendations include the following:

1. *Avoidance of QT-prolonging medications:*
Avoid certain antibiotics, antipsychotics, and antiarrhythmic drugs^161^
Consult with physicians or pharmacists before initiating new medications
2. *Electrolyte balance:*
Maintain normal potassium and magnesium levels^64^
Avoid diuretics and medications causing electrolyte disturbances
3. *Exercise restrictions:*
Recommendations vary by genetic subtype and risk profile^166^
*LQT1:* Avoid competitive sports and strenuous exercise, especially swimming^167^
*LQT2 and LQT3:* Low-intensity exercise under medical supervision may be allowed^168^
4. *Stress management:*
Emotional stress can trigger arrhythmic events, particularly in LQT1 and LQT2^169^^,^^170^
Implement stress management techniques (eg, relaxation therapy and cognitive-behavioral therapy)^171^
5. *Trigger avoidance:*
Educate patients about genotype-specific triggers
*LQT2:* Avoid sudden loud noises and use gradual wake-up alarms^113^
6. *Compliance with medical therapy:*
Emphasize importance of adhering to prescribed therapy (eg, β-blockers)^111^

Patients should undergo regular follow-up to monitor the QTc interval, assess therapy response, and adjust management plans. They should also be educated about arrhythmic event signs and symptoms, with instructions to seek prompt medical attention for syncope, palpitations, or other concerning symptoms.

#### Pharmacological interventions

##### β-Blockers

β-Blockers are the mainstay of LQTS pharmacological therapy, recommended for all patients with a confirmed diagnosis.^13^^,^^172^ They attenuate sympathetic stimulation effects on the heart, reducing cardiac event risk by up to 60%.^173^ Commonly used β-blockers include propranolol and nadolol, with nadolol showing significant risk reduction in patients with LQT2.^174^ The choice and dosing should be individualized on the basis of genotype, age, weight, and tolerance.^175^, ^176^, ^177^ Patients should be monitored for side effects such as nightmares, coldness of extremities, tiredness, dizziness, and impaired physical condition.^162^

##### Potassium channel openers

Potassium channel openers such as nicorandil and pinacidil have been proposed for LQTS, particularly LQT2.^178^^,^^179^ They enhance outward potassium current (I_Kr_) and shorten APD. However, their use is limited by lack of clinical trial data and side effect concerns.^180^

##### Other antiarrhythmic drugs

Mexiletine and ranolazine have been used in select patients with LQTS intolerant or unresponsive to β-blockers.^74^^,^^181^ Mexiletine, a class IB antiarrhythmic, shortens QT interval and reduces arrhythmic events in LQT3.^182^ Ranolazine, an I_Na__L_ inhibitor, shows benefits in LQT3 and aLQTS.^13^^,^^183^^,^^184^ These medications are currently off-label and reserved for patients failing first-line therapies.^185^

##### Role of magnesium and potassium supplements

Magnesium and potassium supplements are used as adjunctive therapies in LQTS, particularly for recurrent arrhythmic events despite β-blocker therapy.^63^^,^^186^ Magnesium does not shorten QT interval but can reduce TdP risk in aLQTS.^187^ Potassium supplements help maintain normal serum levels, preventing QT prolongation and arrhythmic events.^13^^,^^188^ Routine use is not currently recommended; their use should be individualized on the basis of electrolyte status and clinical response.

#### Device therapy

##### ICDs

ICDs are recommended for patients with LQTS who have survived a cardiac arrest or have recurrent syncope despite optimal medical therapy.^129^ They detect and terminate ventricular arrhythmias, such as TdP and ventricular fibrillation, by delivering an electric shock. The decision to implant an ICD should be based on a careful assessment of the patient’s SCD risk and the potential benefits and risks of the device.^189^ Patients with ICDs require regular follow-up to monitor device function, assess shock appropriateness, and adjust programming as needed.^190^

##### Pacemakers

Pacemakers may be considered for patients with LQTS with bradycardia-induced QT prolongation or recurrent syncope despite β-blocker therapy.^191^ They maintain a stable heart rate and prevent bradycardia, which can trigger arrhythmic events in patients with LQTS.^192^ The decision to implant a pacemaker should be individualized on the basis of the patient’s clinical presentation, ECG findings, and response to medical therapy. In some cases, a dual-chamber pacemaker may be preferred to optimize atrioventricular synchrony and prevent pacemaker-induced QT prolongation.^193^

#### Surgical and other interventions

##### LCSD

LCSD is a surgical procedure involving the removal of the left stellate ganglion and the first 3–4 thoracic ganglia, responsible for sympathetic innervation of the heart.^194^ It has been shown to reduce arrhythmic events and SCD risk in patients with LQTS intolerant or unresponsive to β-blockers.^195^ LCSD works by decreasing sympathetic tone and attenuating adrenergic stimulation effects on the heart. It is typically reserved for high-risk patients or those with recurrent events despite optimal medical therapy.^196^ Complications include Horner syndrome, hemothorax, and pneumothorax, but these are generally rare and self-limited.^197^

##### Catheter ablation

Catheter ablation has been proposed as a potential therapy for patients with LQTS with recurrent arrhythmic events despite optimal medical and device therapy.^198^ The procedure involves identifying and ablating focal triggers or substrates responsible for initiating or maintaining ventricular arrhythmias.^199^ However, its role in LQTS is currently limited by lack of clinical trial data and concerns about procedural risks, such as heart block and perforation. Catheter ablation should be considered only in highly selected patients and performed by experienced operators in specialized centers.

##### Experimental therapies and future directions

Several experimental therapies are being investigated for LQTS treatment^17^^,^^200^, ^201^, ^202^: Gene therapy aims to correct the underlying genetic defect by delivering normal copies of the affected gene to cardiac cells using viral vectors. Stem cell therapy involves using pluripotent stem cells to regenerate or replace dysfunctional cardiac tissue.^203^ Novel pharmacological agents, such as allosteric modulators of ion channels and gene-specific therapies, are being developed to target specific LQTS subtypes and improve the specificity and efficacy of treatment.^204^

In addition to these experimental therapies, future directions in LQTS research include the development of more accurate risk stratification tools, such as genotype-specific risk scores and machine learning algorithms.^205^^,^^206^ These tools may help identify patients at the highest risk of SCD and guide personalized management strategies.^207^ Other areas of active research include the identification of novel genetic and epigenetic modifiers of LQTS, the elucidation of genotype-phenotype correlations, and the development of more sensitive and specific diagnostic tests.^208^

### Future directions in LQTS research

The field of LQTS research has made significant strides in recent years, with advancements in genetic testing, risk stratification, and personalized therapies. However, there are still many unanswered questions and areas for future investigation.

#### Advances in genetic testing and personalized medicine

Next-generation sequencing technologies have revolutionized the field of genetic testing, allowing for the rapid and cost-effective analysis of multiple genes simultaneously.^209^ The use of next-generation sequencing in LQTS has led to the identification of novel disease-causing variants and the expansion of the genetic spectrum of the disorder.^210^ Future research should focus on the clinical validation and interpretation of these variants as well as the development of standardized guidelines for the reporting of genetic test results.^211^

The integration of genetic information with clinical and electrophysiological data has the potential to improve risk stratification and guide personalized treatment strategies.^212^ For example, the use of induced pluripotent stem cell–derived cardiomyocytes from patients with LQTS has emerged as a promising tool for drug screening and precision medicine.^213^ Future studies should explore the utility of induced pluripotent stem cell–derived cardiomyocytes in predicting patient-specific responses to therapies and identifying novel drug targets.^214^

#### Novel pharmacological agents

While β-blockers remain the mainstay of therapy for LQTS, they are not effective in all patients and may have significant side effects.^215^ Therefore, there is a need for novel pharmacological agents that can specifically target the underlying molecular defects in LQTS.^216^^,^^217^ Some promising candidates include I_Na__L_ inhibitors (eg, ranolazine and eleclazine), potassium channel openers (eg, nicorandil and retigabine), and calcium channel blockers (eg, verapamil).^218^, ^219^, ^220^ Future research should focus on the preclinical and clinical evaluation of these agents as well as the identification of new therapeutic targets on the basis of the expanding knowledge of LQTS pathophysiology.^221^

#### Innovative device technologies

ICDs have been shown to be effective in preventing SCD in patients with high-risk LQTS.^189^ However, ICDs are associated with significant morbidity and may not be suitable for all patients, particularly children and young adults. Future research should focus on the development of less invasive and more patient-friendly device technologies, such as subcutaneous ICDs,^222^ leadless pacemakers, and wearable defibrillators.

The use of mobile health technologies, such as smartphone apps and wearable sensors, has the potential to improve the monitoring and management of patients with LQTS.^223^ These technologies can help track patient symptoms, medication adherence, and QT interval changes, enabling early detection of arrhythmias and timely interventions. However, further validation and integration of these technologies into clinical practice are needed.

Another promising area of research is the development of gene therapy approaches for LQTS. Preclinical studies have demonstrated the feasibility of using viral vectors to deliver wild-type copies of LQTS-associated genes or to silence mutant alleles in animal models.^224^^,^^225^ While these approaches are still in the early stages of development, they hold promise for providing a curative therapy for LQTS in the future.

#### Clinical trials and registries

Clinical trials are essential for evaluating the safety and efficacy of novel therapies for LQTS. Currently, several trials are underway to assess the efficacy of new pharmacological agents, such as the I_Na__L_ inhibitor eleclazine (GS-6615)^226^ and the I_Ks_ activator ML277^227^, in the treatment of LQTS. These trials may provide additional therapeutic options for patients who do not respond adequately to conventional therapies or who experience intolerable side effects.

Registries play a crucial role in advancing the understanding of LQTS by providing valuable data on the natural history, genotype-phenotype correlations, and treatment outcomes in large cohorts of patients. The International LQTS Registry, established in 1979, has been instrumental in defining the clinical characteristics and risk factors for cardiac events in patients with LQTS.^228^ Ongoing efforts to expand and harmonize LQTS registries worldwide will facilitate the identification of novel risk factors, the validation of risk stratification models, and the evaluation of long-term treatment outcomes.

#### Public health strategies and education

Public health strategies and education are crucial for improving the diagnosis, management, and outcomes of LQTS. These strategies may include the following:

Increasing awareness among physicians, nurses, and other relevant medical professionals, educating patients and families, promoting genetic counseling, and implementing school-based screening programs are key components.^142^^,^^229^
Collaborating with patient advocacy groups and community organizations is essential to raise awareness, provide support, and advance research and education efforts.

## Conclusion

Implementation of these strategies and initiatives aims to improve LQTS recognition, management, and outcomes, ultimately reducing the burden of SCD associated with this disorder.

LQTS remains a complex and potentially life-threatening cardiac channelopathy that presents significant challenges in diagnosis, risk stratification, and management. This comprehensive review has highlighted the multifaceted nature of LQTS, encompassing its genetic basis, pathophysiological mechanisms, clinical presentation, and current treatment strategies.

The expanding knowledge of LQTS genetics has led to improved diagnostic capabilities and a better understanding of genotype-phenotype correlations. However, the variable expressivity and incomplete penetrance of LQTS-associated mutations underscore the need for a comprehensive approach to diagnosis and risk assessment that integrates clinical, ECG, and genetic data.

Management of LQTS has evolved significantly, with β-blockers remaining the cornerstone of therapy for most patients. The role of more aggressive interventions, such as LCSD and ICDs, has been further defined for high-risk individuals.

Despite these advances, several challenges persist. The development of more effective and targeted therapies, improvement of risk stratification models, and optimization of management strategies for genotype-specific LQTS subtypes remain active areas of research. The emergence of novel technologies, including gene therapy and personalized medicine approaches, holds promise for future breakthroughs in LQTS treatment.

As we move forward, continued collaborative efforts in research, clinical practice, and public health initiatives will be crucial in improving outcomes for individuals with LQTS. By advancing our understanding of the disorder’s underlying mechanisms and refining our approach to patient care, we can work toward reducing the burden of SCD and improving the quality of life for those affected by LQTS.
